# Ethyl 2-acet­amido-4,5,6,7-tetra­hydro-1-benzothio­phene-3-carboxyl­ate

**DOI:** 10.1107/S160053681202524X

**Published:** 2012-06-13

**Authors:** Asma Mukhtar, M. Nawaz Tahir, Misbahul Ain Khan, Abdul Qayyum Ather, Muhammad Naeem Khan

**Affiliations:** a Institute of Chemistry, University of the Punjab, Lahore, Pakistan; b University of Sargodha, Department of Physics, Sargodha, Pakistan; c Applied Chemistry Research Center, PCSIR Laboratories Complex, Lahore 54600, Pakistan

## Abstract

In the title compound, C_13_H_17_NO_3_S, the dihedral angles between the thio­phene ring and the ethyl ester and acetamide groups are 5.21 (13) and 10.06 (16)°, respectively. The cyclo­hezene ring adopts a half-chair conformation. An *S*(6) ring is formed due to an intra­molecular N—H⋯O hydrogen bond. In the crystal, mol­ecules are linked by C—H⋯O inter­actions between the tetra­hydro-1-benzothio­phene unit and the ethyl ester group, forming *C*(7) chains propagating along the *b*-axis direction.

## Related literature

For related structures, see: Mukhtar *et al.* (2010*a* ▶,*b* ▶).
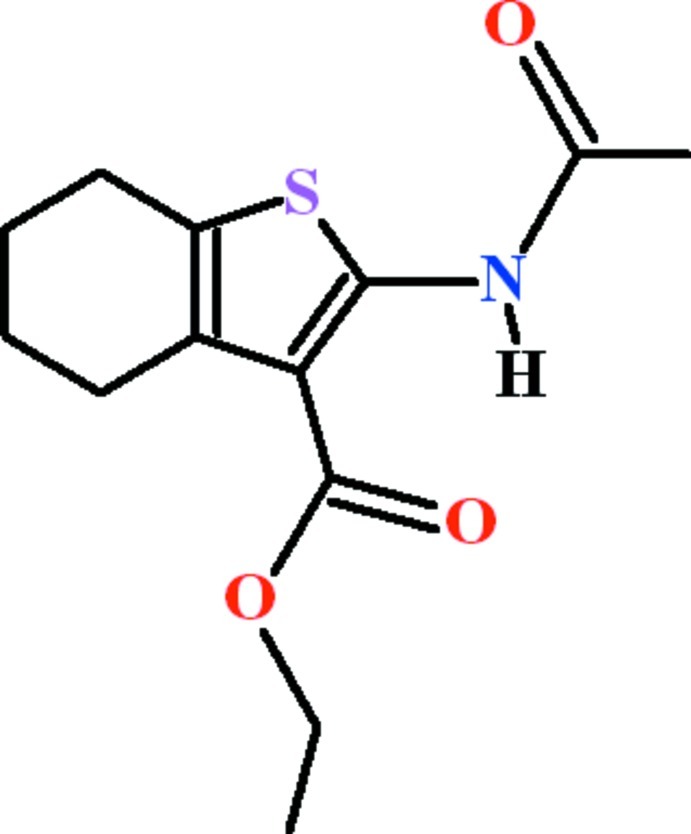


## Experimental

### 

#### Crystal data

C_13_H_17_NO_3_S
*M* *_r_* = 267.34
Monoclinic, 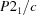

*a* = 10.4267 (4) Å
*b* = 16.6554 (7) Å
*c* = 8.0961 (3) Å
β = 109.610 (1)°
*V* = 1324.43 (9) Å^3^
*Z* = 4
Mo *K*α radiation
μ = 0.24 mm^−1^
*T* = 296 K
0.28 × 0.20 × 0.18 mm

#### Data collection

Bruker Kappa APEXII CCD diffractometer
Absorption correction: multi-scan (*SADABS*; Bruker, 2005 ▶) *T*_min_ = 0.953, *T*_max_ = 0.958
9994 measured reflections
2389 independent reflections
1831 reflections with *I* > 2σ(*I*)
*R*_int_ = 0.032

#### Refinement

*R*[*F* ^2^ > 2σ(*F* ^2^)] = 0.040
*wR*(*F* ^2^) = 0.105
*S* = 1.05
2389 reflections
165 parameters
H-atom parameters constrained
Δρ_max_ = 0.27 e Å^−3^
Δρ_min_ = −0.16 e Å^−3^

### 

Data collection: *APEX2* (Bruker, 2009 ▶); cell refinement: *SAINT* (Bruker, 2009 ▶); data reduction: *SAINT*; program(s) used to solve structure: *SHELXS97* (Sheldrick, 2008 ▶); program(s) used to refine structure: *SHELXL97* (Sheldrick, 2008 ▶); molecular graphics: *ORTEP-3 for Windows* (Farrugia, 1997 ▶) and *PLATON* (Spek, 2009 ▶); software used to prepare material for publication: *WinGX* (Farrugia, 1999 ▶) and *PLATON*.

## Supplementary Material

Crystal structure: contains datablock(s) global, I. DOI: 10.1107/S160053681202524X/hb6836sup1.cif

Structure factors: contains datablock(s) I. DOI: 10.1107/S160053681202524X/hb6836Isup2.hkl

Supplementary material file. DOI: 10.1107/S160053681202524X/hb6836Isup3.cml

Additional supplementary materials:  crystallographic information; 3D view; checkCIF report

## Figures and Tables

**Table 1:** Hydrogen-bond geometry (Å, °)

*D*—H⋯*A*	*D*—H	H⋯*A*	*D*⋯*A*	*D*—H⋯*A*
N1—H1⋯O3	0.86	2.03	2.674 (2)	131
C7—H7*B*⋯O3^i^	0.97	2.50	3.392 (3)	153

Symmetry code: (i) 
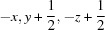
.

**Fig. 1.**
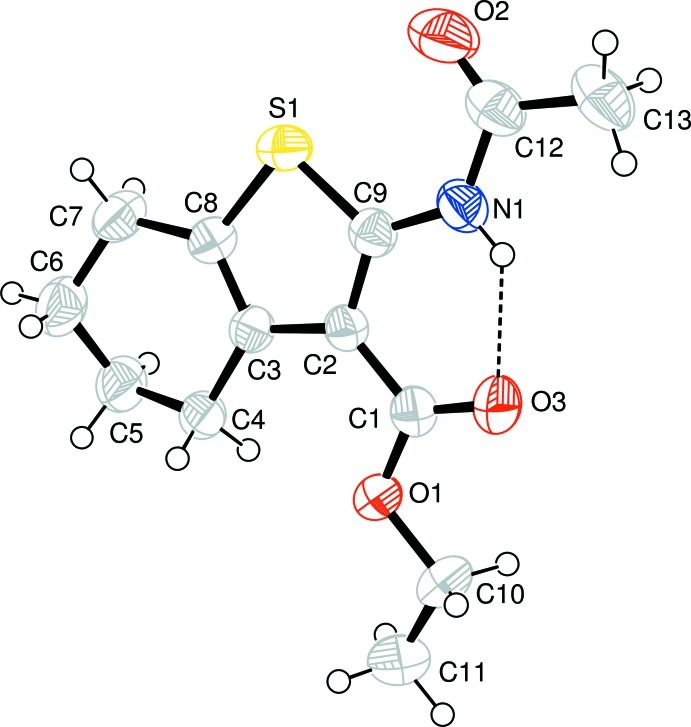
View of the title compound with displacement ellipsoids drawn at the 50% probability level. The dotted line show intramolecular H-bonding.

**Fig. 2.**
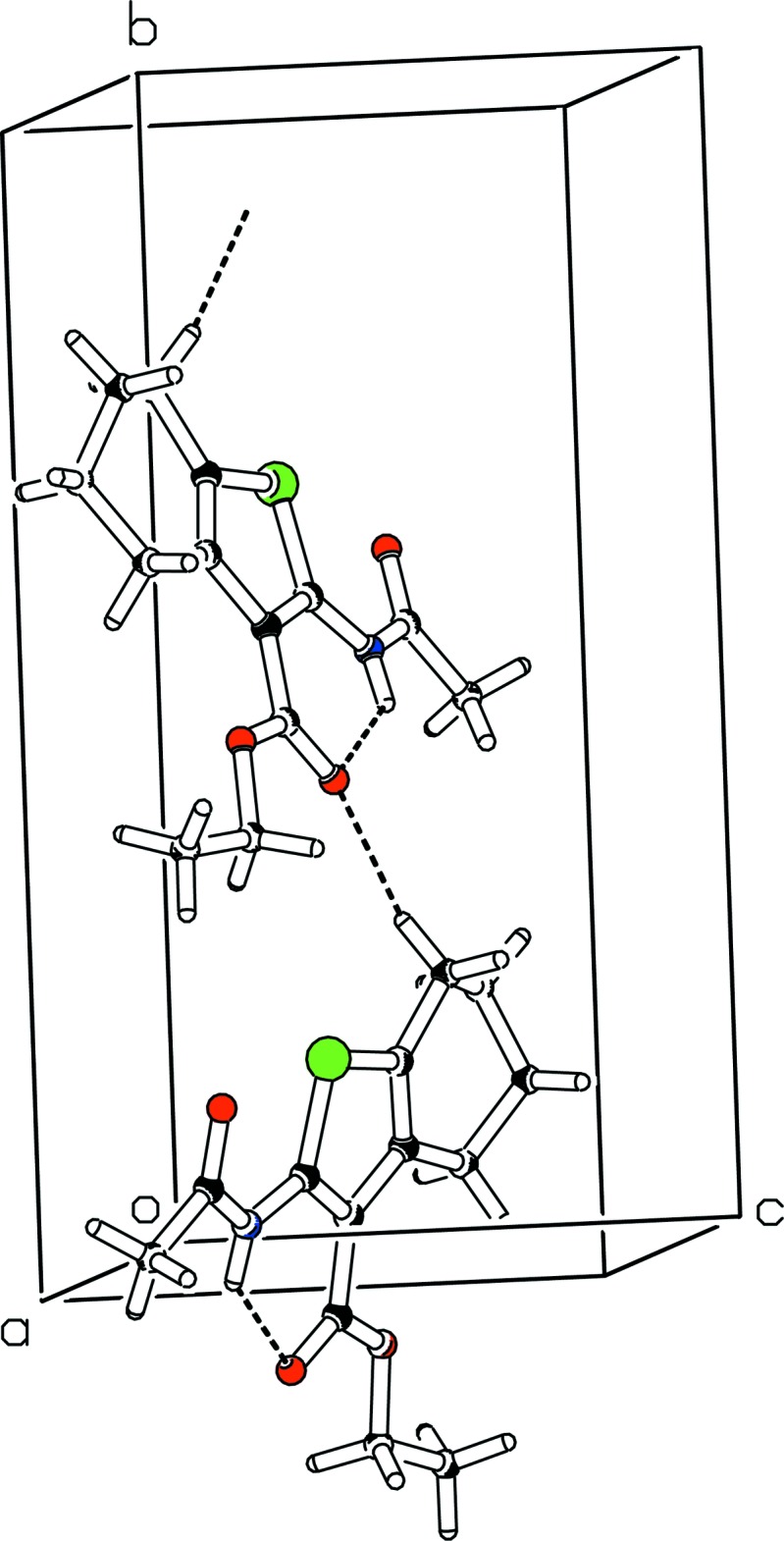
The partial packing, which shows that molecules form C(7) chains extending along the b axis.

## supplementary crystallographic information

### Comment

We reported the crystal structures of ethyl 2-benzamido-4,5,6,7-tetrahydro-1- benzothiophene-3-carboxylate (Mukhtar *et al.*, 2010*a*) and diethyl 5-acetamido-3-methylthiophene-2,4-dicarboxylate (Mukhtar *et al.*, 2010*b*) which are related to the tile compound (I), (Fig. 1).

In (I), the thiophene ring A (S1/C8/C3/C2/C9), ethyl ester group B (O1/C1/O3/C10/C11) and acetamide moiety C (N1/C12/O2/C13) are planar with r. m. s. deviation of 0.0034, 0.0560 and 0.0029 Å, respectively. The dihedral angle between A/B, A/C and B/C is 5.21 (13), 5.17 (14) and 10.06 (16)°, respectively. In the title compound an S(6) ring motif is formed due to intramolecular H-bonding of N—H···O type (Table 1, Fig. 1). The molecules are linked in the form of C(7) chains extending along the [010] direction due to C—H···O type of H-bonding.

### Experimental

Ethyl 2-amino-4,5,6,7-tetrahydrobenzothiophene-3-carboxylate (0.3 g, 1 mmol) was dissolved in chloroform and in this solution 1 ml of acetyl chloride was added. The reaction mixture was refluxed for 8 h. The solvent was removed and the residue was recrystallized by ethanol to get colorless prisms of (I). m.p. 383 K, yield: 0.24 g, 85%.

### Refinement

The H-atoms were positioned geometrically (N—H = 0.86, C–H = 0.96–0.97 Å) and refined as riding with *U*_iso_(H) = *xU*_eq_(C, N), where *x* = 1.5 for methyl and *x* = 1.2 for other H-atoms.

### Crystal data

**Table:**

C_13_H_17_NO_3_S	*F*(000) = 568
*M**_r_* = 267.34	*D*_x_ = 1.341 Mg m^−^^3^
Monoclinic, *P*2_1_/*c*	Mo *K*α radiation, λ = 0.71073 Å
Hall symbol: -P 2ybc	Cell parameters from 1831 reflections
*a* = 10.4267 (4) Å	θ = 2.4–25.3°
*b* = 16.6554 (7) Å	µ = 0.24 mm^−^^1^
*c* = 8.0961 (3) Å	*T* = 296 K
β = 109.610 (1)°	Prism, colorless
*V* = 1324.43 (9) Å^3^	0.28 × 0.20 × 0.18 mm
*Z* = 4	

### Data collection

**Table:**

Bruker Kappa APEXII CCD diffractometer	2389 independent reflections
Radiation source: fine-focus sealed tube	1831 reflections with *I* > 2σ(*I*)
Graphite monochromator	*R*_int_ = 0.032
Detector resolution: 8.10 pixels mm^-1^	θ_max_ = 25.3°, θ_min_ = 2.4°
ω scans	*h* = −12→12
Absorption correction: multi-scan (*SADABS*; Bruker, 2005)	*k* = −19→19
*T*_min_ = 0.953, *T*_max_ = 0.958	*l* = −9→9
9994 measured reflections	

### Refinement

**Table:**

Refinement on *F*^2^	Primary atom site location: structure-invariant direct methods
Least-squares matrix: full	Secondary atom site location: difference Fourier map
*R*[*F*^2^ > 2σ(*F*^2^)] = 0.040	Hydrogen site location: inferred from neighbouring sites
*wR*(*F*^2^) = 0.105	H-atom parameters constrained
*S* = 1.05	*w* = 1/[σ^2^(*F*_o_^2^) + (0.0403*P*)^2^ + 0.4757*P*] where *P* = (*F*_o_^2^ + 2*F*_c_^2^)/3
2389 reflections	(Δ/σ)_max_ < 0.001
165 parameters	Δρ_max_ = 0.27 e Å^−^^3^
0 restraints	Δρ_min_ = −0.16 e Å^−^^3^

### Special details

**Table:**

Geometry. Bond distances, angles *etc*. have been calculated using the rounded fractional coordinates. All su's are estimated from the variances of the (full) variance-covariance matrix. The cell e.s.d.'s are taken into account in the estimation of distances, angles and torsion angles
Refinement. Refinement of *F*^2^ against ALL reflections. The weighted *R*-factor *wR* and goodness of fit *S* are based on *F*^2^, conventional *R*-factors *R* are based on *F*, with *F* set to zero for negative *F*^2^. The threshold expression of *F*^2^ > σ(*F*^2^) is used only for calculating *R*-factors(gt) *etc*. and is not relevant to the choice of reflections for refinement. *R*-factors based on *F*^2^ are statistically about twice as large as those based on *F*, and *R*- factors based on ALL data will be even larger.

### Fractional atomic coordinates and isotropic or equivalent isotropic displacement parameters (Å^2^)

**Table:**

	*x*	*y*	*z*	*U*_iso_*/*U*_eq_	
S1	−0.07735 (6)	0.14705 (4)	0.26031 (7)	0.0539 (2)	
O1	0.22774 (14)	−0.08502 (9)	0.4124 (2)	0.0570 (5)	
O2	−0.31724 (17)	0.09817 (13)	0.0095 (2)	0.0848 (8)	
O3	0.03140 (16)	−0.11247 (10)	0.2017 (2)	0.0646 (6)	
N1	−0.15117 (16)	0.00537 (11)	0.0871 (2)	0.0514 (6)	
C1	0.1049 (2)	−0.06486 (13)	0.3047 (3)	0.0471 (7)	
C2	0.06878 (18)	0.01882 (12)	0.3219 (2)	0.0408 (6)	
C3	0.14704 (19)	0.07906 (12)	0.4432 (2)	0.0414 (6)	
C4	0.2851 (2)	0.06876 (13)	0.5811 (3)	0.0488 (7)	
C5	0.3212 (3)	0.13991 (14)	0.7081 (3)	0.0653 (8)	
C6	0.2832 (3)	0.21870 (14)	0.6192 (3)	0.0690 (9)	
C7	0.1317 (2)	0.22493 (13)	0.5254 (3)	0.0591 (8)	
C8	0.0810 (2)	0.14974 (12)	0.4223 (3)	0.0459 (7)	
C9	−0.05484 (19)	0.04848 (13)	0.2159 (3)	0.0444 (7)	
C10	0.2653 (3)	−0.16924 (14)	0.4095 (4)	0.0742 (10)	
C11	0.3917 (3)	−0.18249 (18)	0.5583 (4)	0.0926 (13)	
C12	−0.2783 (2)	0.03142 (17)	−0.0096 (3)	0.0592 (9)	
C13	−0.3644 (2)	−0.02882 (17)	−0.1363 (3)	0.0729 (9)	
H1	−0.12894	−0.04227	0.06581	0.0616*	
H4A	0.35340	0.06365	0.52467	0.0585*	
H4B	0.28594	0.01977	0.64624	0.0585*	
H5A	0.27501	0.13359	0.79324	0.0783*	
H5B	0.41841	0.13940	0.77120	0.0783*	
H6A	0.33036	0.22575	0.53537	0.0828*	
H6B	0.31193	0.26133	0.70545	0.0828*	
H7A	0.08553	0.23261	0.61017	0.0709*	
H7B	0.11222	0.27086	0.44724	0.0709*	
H10A	0.27966	−0.18170	0.29995	0.0889*	
H10B	0.19331	−0.20356	0.42015	0.0889*	
H11A	0.37805	−0.16655	0.66514	0.1389*	
H11B	0.46381	−0.15119	0.54198	0.1389*	
H11C	0.41548	−0.23836	0.56466	0.1389*	
H13A	−0.41082	−0.00295	−0.24610	0.1095*	
H13B	−0.43007	−0.05116	−0.08977	0.1095*	
H13C	−0.30755	−0.07092	−0.15408	0.1095*	

### Atomic displacement parameters (Å^2^)

**Table:**

	*U*^11^	*U*^22^	*U*^33^	*U*^12^	*U*^13^	*U*^23^
S1	0.0487 (3)	0.0506 (4)	0.0574 (4)	0.0098 (3)	0.0112 (3)	0.0046 (3)
O1	0.0475 (8)	0.0389 (9)	0.0741 (10)	0.0041 (7)	0.0066 (7)	−0.0051 (7)
O2	0.0593 (11)	0.0900 (15)	0.0849 (13)	0.0170 (10)	−0.0024 (9)	−0.0038 (11)
O3	0.0608 (10)	0.0489 (10)	0.0724 (10)	−0.0073 (8)	0.0068 (8)	−0.0142 (8)
N1	0.0423 (10)	0.0563 (12)	0.0496 (10)	−0.0041 (8)	0.0077 (8)	−0.0017 (9)
C1	0.0448 (12)	0.0451 (13)	0.0505 (12)	−0.0040 (10)	0.0148 (10)	−0.0003 (10)
C2	0.0381 (10)	0.0407 (12)	0.0439 (11)	−0.0022 (9)	0.0142 (8)	0.0012 (9)
C3	0.0441 (11)	0.0401 (12)	0.0407 (10)	−0.0008 (9)	0.0152 (9)	0.0028 (9)
C4	0.0471 (12)	0.0443 (12)	0.0481 (12)	−0.0007 (9)	0.0069 (9)	0.0002 (10)
C5	0.0655 (15)	0.0544 (15)	0.0590 (14)	−0.0056 (12)	−0.0015 (12)	−0.0052 (12)
C6	0.0776 (17)	0.0502 (15)	0.0660 (15)	−0.0082 (12)	0.0067 (13)	−0.0079 (12)
C7	0.0723 (16)	0.0418 (14)	0.0577 (13)	0.0061 (11)	0.0147 (12)	−0.0016 (10)
C8	0.0488 (12)	0.0424 (12)	0.0461 (11)	0.0033 (9)	0.0154 (9)	0.0028 (10)
C9	0.0428 (11)	0.0465 (12)	0.0448 (11)	−0.0016 (9)	0.0159 (9)	0.0035 (9)
C10	0.0717 (17)	0.0403 (14)	0.103 (2)	0.0095 (12)	0.0192 (15)	−0.0085 (13)
C11	0.0626 (17)	0.0620 (18)	0.139 (3)	0.0162 (14)	0.0149 (18)	0.0113 (18)
C12	0.0469 (13)	0.0751 (18)	0.0500 (13)	−0.0002 (12)	0.0087 (10)	0.0071 (12)
C13	0.0527 (14)	0.096 (2)	0.0562 (14)	−0.0106 (14)	0.0001 (11)	0.0007 (14)

### Geometric parameters (Å, º)

**Table:**

S1—C8	1.731 (2)	C10—C11	1.474 (4)
S1—C9	1.714 (2)	C12—C13	1.499 (4)
O1—C1	1.328 (3)	C4—H4A	0.9700
O1—C10	1.459 (3)	C4—H4B	0.9700
O2—C12	1.211 (3)	C5—H5A	0.9700
O3—C1	1.218 (3)	C5—H5B	0.9700
N1—C9	1.382 (3)	C6—H6A	0.9700
N1—C12	1.364 (3)	C6—H6B	0.9700
N1—H1	0.8600	C7—H7A	0.9700
C1—C2	1.463 (3)	C7—H7B	0.9700
C2—C9	1.378 (3)	C10—H10A	0.9700
C2—C3	1.449 (3)	C10—H10B	0.9700
C3—C4	1.506 (3)	C11—H11A	0.9600
C3—C8	1.346 (3)	C11—H11B	0.9600
C4—C5	1.531 (3)	C11—H11C	0.9600
C5—C6	1.485 (3)	C13—H13A	0.9600
C6—C7	1.509 (4)	C13—H13B	0.9600
C7—C8	1.499 (3)	C13—H13C	0.9600
			
C8—S1—C9	91.19 (10)	C4—C5—H5A	109.00
C1—O1—C10	115.95 (19)	C4—C5—H5B	109.00
C9—N1—C12	126.0 (2)	C6—C5—H5A	109.00
C9—N1—H1	117.00	C6—C5—H5B	109.00
C12—N1—H1	117.00	H5A—C5—H5B	108.00
O1—C1—O3	122.1 (2)	C5—C6—H6A	109.00
O3—C1—C2	124.3 (2)	C5—C6—H6B	109.00
O1—C1—C2	113.63 (18)	C7—C6—H6A	109.00
C1—C2—C9	119.95 (18)	C7—C6—H6B	109.00
C1—C2—C3	128.32 (17)	H6A—C6—H6B	108.00
C3—C2—C9	111.72 (18)	C6—C7—H7A	110.00
C2—C3—C8	111.88 (17)	C6—C7—H7B	110.00
C2—C3—C4	127.17 (18)	C8—C7—H7A	110.00
C4—C3—C8	120.95 (18)	C8—C7—H7B	110.00
C3—C4—C5	111.59 (19)	H7A—C7—H7B	108.00
C4—C5—C6	113.14 (19)	O1—C10—H10A	110.00
C5—C6—C7	111.6 (2)	O1—C10—H10B	110.00
C6—C7—C8	109.67 (18)	C11—C10—H10A	110.00
C3—C8—C7	126.2 (2)	C11—C10—H10B	110.00
S1—C8—C3	112.99 (16)	H10A—C10—H10B	108.00
S1—C8—C7	120.80 (16)	C10—C11—H11A	109.00
N1—C9—C2	125.09 (19)	C10—C11—H11B	109.00
S1—C9—N1	122.70 (16)	C10—C11—H11C	109.00
S1—C9—C2	112.22 (16)	H11A—C11—H11B	109.00
O1—C10—C11	107.6 (2)	H11A—C11—H11C	109.00
N1—C12—C13	115.0 (2)	H11B—C11—H11C	109.00
O2—C12—N1	121.4 (2)	C12—C13—H13A	109.00
O2—C12—C13	123.5 (2)	C12—C13—H13B	109.00
C3—C4—H4A	109.00	C12—C13—H13C	109.00
C3—C4—H4B	109.00	H13A—C13—H13B	109.00
C5—C4—H4A	109.00	H13A—C13—H13C	110.00
C5—C4—H4B	109.00	H13B—C13—H13C	109.00
H4A—C4—H4B	108.00		
			
C9—S1—C8—C3	−0.74 (18)	C9—C2—C3—C4	178.71 (19)
C9—S1—C8—C7	−179.82 (19)	C9—C2—C3—C8	−0.6 (2)
C8—S1—C9—N1	−179.31 (19)	C1—C2—C9—S1	179.01 (15)
C8—S1—C9—C2	0.37 (17)	C1—C2—C9—N1	−1.3 (3)
C10—O1—C1—O3	−4.5 (3)	C3—C2—C9—S1	0.0 (2)
C10—O1—C1—C2	175.9 (2)	C3—C2—C9—N1	179.72 (19)
C1—O1—C10—C11	−169.7 (2)	C2—C3—C4—C5	−167.96 (19)
C12—N1—C9—S1	−5.9 (3)	C8—C3—C4—C5	11.3 (3)
C12—N1—C9—C2	174.4 (2)	C2—C3—C8—S1	0.9 (2)
C9—N1—C12—O2	1.2 (4)	C2—C3—C8—C7	179.9 (2)
C9—N1—C12—C13	−177.8 (2)	C4—C3—C8—S1	−178.48 (15)
O1—C1—C2—C3	−2.4 (3)	C4—C3—C8—C7	0.6 (3)
O1—C1—C2—C9	178.80 (19)	C3—C4—C5—C6	−41.8 (3)
O3—C1—C2—C3	178.0 (2)	C4—C5—C6—C7	61.4 (3)
O3—C1—C2—C9	−0.8 (3)	C5—C6—C7—C8	−46.2 (3)
C1—C2—C3—C4	−0.2 (3)	C6—C7—C8—S1	−164.26 (17)
C1—C2—C3—C8	−179.5 (2)	C6—C7—C8—C3	16.8 (3)

### Hydrogen-bond geometry (Å, º)

**Table:**

*D*—H···*A*	*D*—H	H···*A*	*D*···*A*	*D*—H···*A*
N1—H1···O3	0.86	2.03	2.674 (2)	131
C7—H7*B*···O3^i^	0.97	2.50	3.392 (3)	153

Symmetry code: (i) −*x*, *y*+1/2, −*z*+1/2.
